# Prognosis of Vascular Access in Haemodialysis Patients with Autosomal Dominant Polycystic Kidney Disease

**DOI:** 10.1038/s41598-020-58441-5

**Published:** 2020-02-06

**Authors:** Tsung-Lun Lee, Chun-Fan Chen, Ann Charis Tan, Chia-Hao Chan, Shuo-Ming Ou, Fan-Yu Chen, Ko-Wen Yu, Yung-Tai Chen, Chih-Ching Lin

**Affiliations:** 1 School of Medicine, National Yang-Ming University, Taipei, Taiwan; 2 Division of Nephrology, Department of Medicine, Taipei Veterans General Hospital, Taipei, Taiwan; 3 Department of Nephrology, Fooyin University Hospital, Pingtung, Taiwan; 4 Division of Nephrology, Department of Internal Medicine, National Yang-Ming University Hospital, Yilan, Taiwan; 5 Division of Nephrology, Department of Internal Medicine, Taipei City Hospital, Heping Fuyou Branch, Taipei, Taiwan

**Keywords:** Polycystic kidney disease, Haemodialysis

## Abstract

Vascular diseases are commonly observed in patients with autosomal dominant polycystic kidney disease (ADPKD). We aim to investigate the differences in the risk for arteriovenous fistula or graft (AVF/AVG) dysfunction in haemodialysis (HD) patients with and without ADPKD. 557 ADPKD and 1671 non-ADPKD patients were enrolled in the study after propensity score matching. The primary outcome measure is the incidence rate of AVF/AVG dysfunction. The incidence rates and risks of AVF/AVG dysfunction (per 100 person-years) for ADPKD and non-ADPKD patients were (1) 38.83 and 48.99 [SHR = 0.79, P = 0.137], respectively, for within 90 days, (2) 45.85 and 51.31 [SHR = 0.90, P = 0.300], respectively, for within 180 days, (3) 44.42 and 41.40 [SHR = 1.08, P = 0.361], respectively, for within the first year, (4) 27.38 and 24.69 [SHR = 1.09, P = 0.168], respectively, for within 5 years, (5) 17.35 and 13.80 [SHR = 1.19, P = 0.045], respectively, for between the 1st and 10th year, and (6) 25.40 and 21.22 [SHR = 1.14, P = 0.031], respectively, for all periods. ADPKD patients had lower incidence rates of AVF/AVG dysfunction within the first 180 days than non-ADPKD patients, but presented a higher incidence rate after 1 year of AVF/AVG creation and onwards.

## Introduction

Autosomal dominant polycystic kidney disease (ADPKD) is a common hereditary kidney disease that has affected 12.5 million people worldwide^1^. According to the National Health Insurance Research Database (NHIRD) in Taiwan, approximately 2.4% of patients with ADPKD progressed to end-stage renal disease (ESRD) and underwent haemodialysis (HD). Besides, 1.4% of ESRD cases in Taiwan were caused by ADPKD^2–4^.

Abdominal aortic aneurysm (AAA), intracranial aneurysm (ICA), dolichoectasia, and dissections of major vessels are common vascular complications in ADPKD patients in the clinical setting^5,6^. They exhibited a higher prevalence of ICA (4.0–11.7%) than the general population (1.0%)^7,8^. This occurrence can be demonstrated by decreased polycystin-2 (PC2) concentration levels, which leads to the luminal dilatation and the irregular thickening and thinning of the arterial wall in the Pkd2^+/−^ vessels^9^. Both arteriovenous fistula (AVF) and arteriovenous graft (AVG) are modes of vascular access selection recommended by the National Kidney Foundation Kidney Disease Outcomes Quality Initiative guidelines for patients undergoing HD and are widely utilized in Taiwan and internationally. However, the patency of AVF/AVG in ADPKD patients due to the nature of their vasculature abnormality are rarely discussed.

There were limited studies providing information regarding AVF patency in this particular population and even then, these studies had produced conflicting results. Monroy-Cuadros *et al*. performed a retrospective study on 831 HD patients and found that the loss of primary functional patency of the AVF was 18.8% in patients with ADPKD and 8.8% in patients with diabetic nephropathy within a six-month study period^10^. However, Rodriguez *et al*. showed that the frequency of patients needing HD treatment using vascular catheters due to the lack of AVF was 3% and 11% for patients with ADPKD and diabetic nephropathy, respectively^11^.

Taiwan, with a population of 23 million people, has one of the highest incidence and prevalence rates of treated ESRD (476 and 3317 per million population per year, respectively), according to the 2018 United States Renal Data System annual report. The country’s national health insurance system covers almost all of the related expenditure needed for HD and other related treatments, including AVF/AVG creation, percutaneous transluminal angioplasty, and surgical interventions such as thrombectomy. The system offers a good opportunity to establish a large-scale study to analyse the long-term dysfunction rate of AVF/AVG in HD patients.

## Methods

### National health insurance research database

The National Health Insurance Administration has provided compulsory universal health insurance in Taiwan since 1995. All citizens and residents of Taiwan are required to enroll in the program except for prisoners. Through this program, ESRD patients have full coverage for renal replacement therapy. Healthcare institutions are required to submit standard computerized claim documents for renal replacement therapy to the National Health Insurance Administration. The National Health Insurance Research Database, covering almost all (99%) of the inpatient and outpatient medical benefit claims for Taiwan’s 23 million residents, is one of the most comprehensive and largest databases in the world and has been utilized extensively in various studies^2–4^. Patient identification number, gender, birthday, dates of admission and discharge, healthcare institutions providing services, ICD-9-CM and ICD-10-CM diagnostic and procedure codes (up to five each), and outcomes are encrypted. The study was based on the Helsinki Declaration (edition 6, revised 2000) and was approved by the Institutional Review Board of Taipei Veterans General Hospital. The methods were carried out in accordance with the approved guidelines. Informed consent was waived because the dataset was encrypted and de-identified. This study tapped the National Health Insurance Research Database for ambulatory care claims, inpatient claims, and the updated registry for beneficiaries from 2000 to 2012. The primary outcome is the cumulative incidence rate of AVF/AVG dysfunction, which is defined as the need from the time of creation to the first episode of dysfunction with the need for any procedure such as angioplasty, thrombectomy, or creation of another AVF/AVG within 3 months, 1 year, 5 years, and 10 years. Other outcomes included the occurrence of major adverse cardiovascular events (MACE) (the first occurrence of death from cardiovascular causes, nonfatal myocardial infarction or nonfatal stroke), myocardial infarction, and ischemic stroke.

### Patient selection

Data were collected retrospectively for all HD patients from the NHIRD in Taiwan from 2000 to 2012. The patients were divided into two subgroups (ADPKD and non-ADPKD group). The exclusion criteria of the study are as follows: (1) under the age of 20, (2) undergoing peritoneal dialysis, (3) pregnant, (4) kidney transplant recipients, and (5) had never initiated HD via AVF/AVG or installed a permanent double-lumen catheter after AVF/AVG creation. The National Health Insurance Administration issued the catastrophic illness card to HD patients who require life-long renal replacement therapy. Participants who are not eligible for this document were also excluded from the study. In this study, the socioeconomic and clinical characteristics of participants with ADPKD were analysed in comparison with the participants without ADPKD.

### Statistical analysis

SAS version 8.0 (SAS Institute, Cary, North Carolina, USA) was used to conduct data management and statistical analysis. Distributions of continuous variables in groups were expressed as mean ± SD and compared using the t-test. All data were tested for normal distribution. Categorical variables were analysed using the chi-square test. The propensity scores of the likelihood of ADPKD were determined by multivariate logistic regression analysis, conditional on the baseline covariates (Supplementary Table 1). Three non-ADPKD patients were matched with each patient in the ADPKD cohort with a similar propensity score based on the nearest neighbor matching without replacement using calipers of width equal to 0.1 of the standard deviation of the logit of the propensity score. The survival curves for the cumulative incidence rate of AVF/AVG dysfunction were assessed using Cox regression and Kaplan-Meier methods and compared using the log-rank test. All reported tests were two-sided. A statistically significant value was set at P < 0.05.

## Results

There were a total number of 98721 HD participants enrolled during the study period, but 19174 participants were excluded from the analysis for the following reasons: 4268 underwent peritoneal dialysis, 277 were under the age of 20, 0 were pregnant, 417 were kidney transplant recipients, 3096 had never initiated HD via AVF/AVG, and 11533 had installed a permanent double-lumen catheter after AVF/AVG creation.

A total of 79547 patients were selected, which comprised of 1652 ADPKD and 77895 non-ADPKD patients. 557 ADPKD and 27371 non-ADPKD patients remained after the catastrophic illness card exclusion criteria, and a final sample of 557 ADPKD and 1671 non-ADPKD patients remained in the study after propensity score matching. Table 1 shows the baseline characteristics of the enrolled patients. There were no significant differences between ADPKD and non-ADPKD patients in age (mean age: 56 and 55 years old, respectively), gender (282 and 837 males, respectively), Charlson Comorbidity Index scores (mean scores of 4.7 and 4.6, respectively), and the number of patients with AVF (505 and 1516, respectively). The usage of concomitant medications and comorbidities were also similar in both groups. The overall characteristics were not found to be statistically significant after the use of propensity score matching.

**Table 1:** Baseline Characteristics of Patients.

Characteristics	ADPKD Group (N = 557)	Non-ADPKD Group (N = 1671)	P
Age, years, mean (SD)	56.0 (12.6)	55.0 (14.7)	0.920
**Gender**			
Male	282 (50.6)	837 (50.1)	0.826
Female	275 (49.4)	834 (49.9)	
CCI score, mean (SD)	4.7 (2.2)	4.6 (2.2)	0.852
AVF	505 (90.7)	1516 (90.7)	0.966
**Concomitant medications**			
Antiplatelet agents^‡^	148 (26.6)	452 (27.0)	0.825
ACE inhibitor or ARB	225 (40.4)	672 (40.2)	0.940
Beta blocker	283 (50.8)	849 (50.8)	>0.99
Calcium channel blocker	392 (70.4)	1140 (68.2)	0.342
Statin	40 (7.2)	119 (7.1)	0.962
**Comorbidities**			
Diabetes mellitus	157 (28.2)	433 (25.9)	0.292
Hypertension	531 (95.3)	1597 (95.6)	0.813
Myocardial infarction	23 (4.1)	68 (4.1)	0.951
Heart failure	100 (18.0)	305 (18.3)	0.874
Peripheral vascular disease	30 (5.4)	82 (4.9)	0.654
Dementia	15 (2.7)	45 (2.7)	>0.99
Chronic pulmonary disease	219 (39.3)	653 (39.1)	0.920
Dyslipidemia	211 (37.9)	643 (38.5)	0.801
Cerebrovascular disease	150 (26.9)	420 (25.1)	0.400
Valvular heart disease	54 (9.7)	144 (8.6)	0.439
Cancer	96 (17.2)	266 (15.9)	0.466

All data are presented as n (%), unless otherwise indicated.

^‡^Including aspirin, clopidogrel, ticlopidine, and cilostazol.

Abbreviations: ADPKD, autosomal dominant polycystic kidney disease; CCI, Charlson Comorbidity Index; AVF, arteriovenous fistula; ACE, angiotensin-converting enzyme; ARB, angiotensin II receptor blocker; SD, standard deviation.

**Figure 1:**
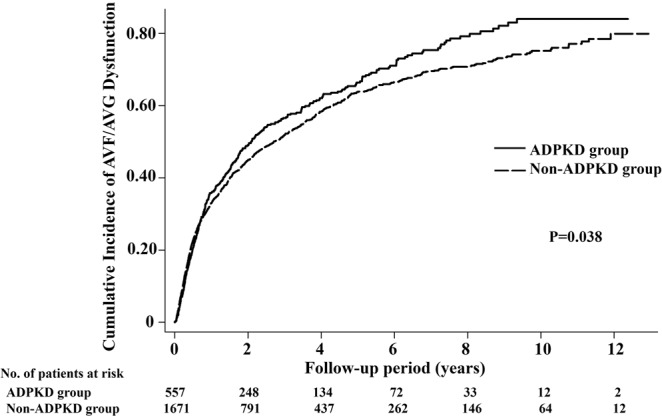
Kaplan-Meier survival estimates showed the cumulative incidence rates of AVF/AVG dysfunction between the ADPKD and non-ADPKD groups over time. There was a statistically significant difference between the two groups (P = 0.038).

Table 2 shows the incidence rates and risks of AVF/AVG dysfunction after propensity score matching was implemented. The incidence rates and risks of AVF/AVG dysfunction (per 100 person-years) for ADPKD and non-ADPKD patients were (1) 38.83 and 48.99 [SHR = 0.79, P = 0.137], respectively, for within 90 days, (2) 45.85 and 51.31 [SHR = 0.90, P = 0.300], respectively, for within 180 days, (3) 44.42 and 41.40 [SHR = 1.08, P = 0.361], respectively, for within the first year, (4) 27.38 and 24.69 [SHR = 1.09, P = 0.168], respectively, for within 5 years, (5) 17.35 and 13.80 [SHR = 1.19, P = 0.045], respectively, for between the 1st and 10th year, and (6) 25.40 and 21.22 [SHR = 1.14, P = 0.031], respectively, for all periods. These findings indicated that ADPKD patients had lower incidence rates of AVF/AVG dysfunction prior to the 1-year follow-up period than non-ADPKD patients. However, the incidence rates of ADPKD patients then became higher than that of non-ADPKD patients after 1 year and reached significant difference in the 1st-year-to-10th-year period and in the overall period. This was also confirmed in the survival curves presenting the cumulative incidence rates of AVF/AVG dysfunction of the ADPKD and non-ADPKD groups in Fig. 1 where the difference between the two groups reached statistical significance (P = 0.038).

**Table 2:** Incidence Rates and Risks of AVF/AVG Dysfunction in ADPKD and Non-ADPKD Groups.

Time Period	ADPKD Group			Non-ADPKD Group (Reference)			Crude		Competing Risk	
	No. of Events	Person- Years	Incidence Rate*	No. of Events	Person- Years	Incidence Rate*	HR (95% CI)	P	SHR (95% CI)	P
All periods	371	1460	25.40	1023	4820	21.22	1.13 (1.01–1.28)	0.038	1.14 (1.01–1.28)	0.031
Within 90 days	51	131	38.83	191	390	48.99	0.79 (0.58–1.08)	0.138	0.79 (0.58–1.08)	0.137
Within 180 days	113	246	45.85	372	725	51.31	0.89 (0.72–1.10)	0.289	0.90 (0.73–1.10)	0.300
First year	197	443	44.42	543	1312	41.40	1.07 (0.91–1.26)	0.418	1.08 (0.92–1.27)	0.361
Within 5 years	335	1223	27.38	946	3832	24.69	1.08 (0.96–1.23)	0.213	1.09 (0.96–1.23)	0.168
1–10 years	174	1003	17.35	473	3428	13.80	1.23 (1.03–1.46)	0.022	1.19 (1.00–1.41)	0.045

^*^Per 10^2^ person-years.

Abbreviations: AVF, arteriovenous fistula; AVG, arteriovenous graft; ADPKD, autosomal dominant polycystic kidney disease; HR, hazard ratio; SHR, subdistribution hazard ratio; CI, confidence interval.

Table 3 shows the incidence rates and risks of AVF/AVG dysfunction in ADPKD and non-ADPKD groups with or without diabetes mellitus. With the non-ADPKD group without diabetes mellitus acting as the reference point, the incidence rates (per 100 person-years) of the non-ADPKD and ADPKD groups without diabetes mellitus were 18.50 and 23.30, respectively. The difference between these two groups were found to be statistically significant (SHR = 1.18, P = 0.015), which indicates that ADPKD may directly affect AVF/AVG dysfunction, even with the exclusion of diabetes as a variable.

**Table 3:** Incidence Rates and Risks of AVF/AVG Dysfunction in ADPKD and Non-ADPKD Groups With or Without Diabetes Mellitus.

Patient Group	No. of Events	Person- Years	Incidence Rate*	Crude		Competing Risk	
				HR (95% CI)	P	SHR (95% CI)	P
Non-ADPKD group without diabetes mellitus	724	3914	18.50	Reference		Reference	
Non-ADPKD group with diabetes mellitus	299	907	32.98	2.87 (1.84–4.49)	<0.001	1.43 (1.25–1.64)	<0.001
ADPKD group without diabetes mellitus	263	1129	23.30	1.18 (0.67–2.06)	0.565	1.18 (1.03–1.36)	0.015
ADPKD group with diabetes mellitus	108	332	32.58	2.60 (1.37–4.94)	0.003	1.43 (1.16–1.76)	0.001

^*^Per 10^2^ person-years.

Abbreviations: AVF, arteriovenous fistula; AVG, arteriovenous graft; ADPKD, autosomal dominant polycystic kidney disease; HR, hazard ratio; SHR, subdistribution hazard ratio; CI, confidence interval.

Table 4 shows the incidence rates and risks of MACE, myocardial infarction, and ischemic stroke in ADPKD and non-ADPKD groups. The incidence rate and risk of MACE (per 100 person-years) for ADPKD and non-ADPKD patients were 1.58 and 1.73 [SHR = 0.92, P = 0.618], respectively. The differences between the two groups were not found to be statistically significant.

**Table 4:** Incidence Rates and Risks of MACE in ADPKD and Non-ADPKD Groups.

Time Period	ADPKD Group			Non-ADPKD Group (Reference)			Crude		Competing Risk	
	No. of Events	Person- Years	Incidence Rate*	No. of Events	Person- Years	Incidence Rate*	HR (95% CI)	P	SHR (95% CI)	P
MACE	44	2790	1.58	143	8252	1.73	0.91 (0.65–1.27)	0.576	0.92 (0.66–1.28)	0.618
Myocardial infarction	19	2848	0.67	72	8431	0.85	0.78 (0.47–1.30)	0.339	0.79 (0.48–1.30)	0.352
Ischemic stroke	29	2832	1.02	79	8360	0.95	1.08 (0.71–1.66)	0.713	1.11 (0.72–1.69)	0.642

^*^Per 10^2^ person-years.

Abbreviations: MACE, major cardiovascular events; AVF, arteriovenous fistula; AVG, arteriovenous graft; ADPKD, autosomal dominant polycystic kidney disease; HR, hazard ratio; SHR, subdistribution hazard ratio; CI, confidence interval.

## Discussion

The primary hypothesis of this study is that there may be a difference observed with regards to AVF/AVG patency due to the genetic variation of vascular complications present in ADPKD, leading to irregular thickening and thinning of the arterial wall and luminal dilatation. We observed that the incidence rate of AVF/AVG dysfunction was lower in the ADPKD group within the 90-day and 180-day follow up period despite not attaining statistical significance. A higher incidence rate of AVF/AVG dysfunction in the ADPKD group was observed from the 1-year follow-up period and onwards. However, the difference only reached statistical significance during the 1-to-10-year and overall follow-up period.

The observation period encompassed in the study was a duration of 10 years, as demonstrated in Table 2 and Fig. 1. The results during the 10-year observation period were not only supported by the findings of Rodriguez *et al*. where there was a lower incidence rate of AVF/AVG dysfunction in early-stage ADPKD, but also by the study of Monroy-Cuadros *et al*. where there was a higher incidence of AVF/AVG dysfunction in later-stage ADPKD. The lower rate of AVF/AVG dysfunction in early-stage ADPKD may be due to vascular wall thinning leading to possible vascular dilatation, while the higher rate of AVF/AVG dysfunction in later-stage ADPKD may be in relation to aneurysmal dilatation, endothelial dysfunction, carotid intima-media thickness, arterial stiffness, oxidative stress, inflammation, and hypertension, as commonly seen in ADPKD patients.

In terms of aneurysmal complications, AAA is a major extrarenal complication observed in ADPKD patients in the clinical setting. A study by Kato *et al*. revealed that the prevalence of AAA in the ADPKD group was 7.1%, which was higher than the other non-ADPKD groups^12^. Palestini *et al*. investigated the incidence of AAA in patients with ESRD undergoing chronic HD and found out that 11 (8.5%) out of the 129 patients have AAA. AAA existed in 19.3% (6/31) of patients with ADPKD and in 5.1% (5/98) of patients with renal insufficiency due to other pathologies^13^. However, a study by Torra *et al*. showed no evidence of a wider aortic diameter or a higher prevalence of AAA in ADPKD patients in any age group^5^. ICA is also another complication observed in ADPKD patients. A greater prevalence rate of ICA was observed, approximately 4% to 11.7%, in ADPKD patients than the general population^7,8^.

The etiology of these vascular abnormalities were investigated by Qian *et al*. in an animal model study where findings exhibited decreased PC2 expression in *Pkd2* ^+^ vessels and an enhanced level of intracranial vascular abnormalities in *Pkd2*^+/−^ mice when smooth muscles have significantly altered intracellular Ca^2+^ homeostasis, which leads to luminal dilatation and irregular thickening and thinning of the arterial wall in *Pkd2*^+/−^ vessels. Polycystin-1 (PC1) and PC2 are membrane-associated proteins encoded by *PKD1* and *PKD2* genes^14,15^. PC2 is a Ca^2+^-permeable channel that can interact with and was regulated by PC1^16,17^. A study by Rossetti *et al*. showed that patients with *PKD1* and *PKD2* mutations were associated with a high risk of ICA^18^.

The *PKD1* mutation position is also an important prognostic factor that determines the likelihood of a patient developing an aneurysm where 5’ mutations are more commonly associated with vascular diseases. Homozygous *PKD1* and *PKD2* mutations in mouse embryo show an expression of polycystins in vascular smooth muscle cells and disorder such as vascular leakage and multiple focal haemorrhages were observed and is a direct factor in ADPKD-associated vascular disease^19^.

The risk of AVF/AVG dysfunction in patients with later-stage ADPKD may be attributed to endothelial dysfunction, carotid intima-media thickness, and arterial stiffness. Kocaman *et al*. demonstrated that there was increased carotid intima–media thickness and significant endothelial dysfunction in both hypertensive and normotensive patients with ADPKD. Endothelial-dependent dilation was significantly worse in hypertensive patients with ADPKD compared to patients with essential hypertension (9.1% ± 4.1% vs. 12.4% ± 4.6%, respectively) and also in normotensive patients with ADPKD compared to healthy subjects (13.1% ± 5.2% vs. 18.1% ± 8.1%, respectively). Moreover, carotid intima-media thickness was significantly greater in both hypertensive (0.71 ± 0.10 mm) and normotensive (0.57 ± 0.14 mm) patients with ADPKD compared with healthy subjects (0.45 ± 0.10 mm)^20^. Borresen *et al*. investigated arterial stiffness in early ADPKD by pulse wave analysis and pulse-wave velocity measurement. The study showed that the reflection of the pulse wave was amplified in young normotensive ADPKD patients, indicating early pathology in the arterial system^21^. Both studies revealed that arterial stiffness starts very early in the course of ADPKD and may contribute to the loss of AVF/AVG patency in our study.

A recent study by Nowak *et al*. testified that arterial stiffness, vascular oxidative stress, and inflammation develop with ADPKD. Brachial artery flow-mediated dilation increased significantly after acute infusion of ascorbic acid in participants with early-stage ADPKD. The endothelial cell protein expression of NF-kB was also greater in this particular group of participants^22^.

Hypertension and ADPKD are closely associated with each other because of the renal cyst enlargement that may stimulate both the circulating and intrarenal renin-angiotensin-aldosterone system^23^. A systematic review conducted by Cagnazzo *et al*. in 563 patients with ADPKD and hypertension showed that hypertension was present in 79.3% of patients with ADPKD, the prevalence of unruptured aneurysms was at 11.5%, and the mean size of ruptured aneurysms was slightly higher than unruptured aneurysms (6 mm vs. 4.4 mm)^24^. A study on blood pressure parameters obtained by ambulatory blood pressure monitoring showed that ADPKD patients with ICA have higher nighttime maximum diastolic blood pressure, higher maximum increases in nighttime diastolic blood pressure from measurement to measurement, and higher standard deviation of the daytime mean arterial pressure compared to those without ICA^25^.

This study has several limitations. This is a retrospective study, conducted in a single country, and the participants were of Chinese ethnicity. The association between ADPKD and non-ADPKD groups with different ethnicities is unknown. On the side of the patients, there are variables such as blood pressure and biochemical data that can be taken into account in the future. On the side of the healthcare professionals involved in the creation and maintenance of vascular access, variables such as the technique and experience of the surgeon during vascular access procedures (the results of a 2010 study using data generated by the Dialysis Outcomes and Practice Patterns Study that enrolled HD patients from 12 countries showed that there was a 34% lower risk of initial fistula failure by surgeons who had created a minimum of 25 fistulas during training^26^), the cannulation skill of the nurse, and the cannulation procedures that may differ in every hospital and clinic, all may have an effect on vascular access patency. However, such variables are hard to account for in database analysis.

In this study, the number of patients with AVG listed in the database were too few. Therefore, it was combined with the number of patients with AVF for the analysis. Although there were no official data analysing the ratio of vascular access types in Taiwan, AVF is the most common form of vascular access for HD in Taiwan based on clinical experience, owing to its lower risk of infection and thrombosis. Nevertheless, there were some data recorded on previous studies. In a study conducted by Chen *et al*. with a total of 42244 patients enrolled in this study, information retrieved from the National Health Insurance Research Database revealed that 89.4% of patients used AVF compared to only 10.6% patients who used AVG during their first long-term dialysis from 2001 to 2006^27^. In another study conducted by Chen *et al*. in 2014 which enrolled 5161 patients on maintenance HD from 25 dialysis centers in Taiwan during 2008–2012, up to 75% of patients use AVF as their vascular access, whereas only 20% and 5% of patients use AVG and tunneled dialysis catheter, respectively^28^. Despite these limitations, our study has enrolled the largest number of ADPKD patients in terms of vascular access analysis and is the first study that has the longest observational period of up to 10 years. The data was obtained from the NHIRD in Taiwan and the results were analysed after adjustment with propensity score matching to eliminate bias between groups.

In conclusion, there was a higher incidence rate of AVF/AVG dysfunction in ADPKD patients during the long-term follow-up period (after 1 year and onwards) but not within a short period of time (within 180 days and shorter). Additional randomized large-scale prospective studies should be conducted the future to confirm the observations made in this study.

### Statement of human rights

All procedures performed in studies involving human participants were in accordance with the ethical standards of the Institutional Review Board of Taipei Veterans General Hospital (2018–02–009BC) and with the 1964 Helsinki declaration and its later amendments or comparable ethical standards. The methods were carried out in accordance with the approved guidelines. Informed consent was waived because the dataset was encrypted and de-identified.

## Supplementary information

Supplementary Table S1.
