# Supplementary material for: Prognosis of Vascular Access in Haemodialysis Patients with Autosomal Dominant Polycystic Kidney Disease
Source: Sci Rep. 2020 Feb 6;10:1985. doi: 10.1038/s41598-020-58441-5 (PMC7004976; doi:10.1038/s41598-020-58441-5)
Supplement: Supplementary file 1 — Supplementary Table S1. [file 41598_2020_58441_MOESM1_ESM.docx]

**Prognosis of Vascular Access in Haemodialysis Patients with Autosomal Dominant Polycystic Kidney Disease**

Tsung-Lun Lee^1,2^, Chun-Fan Chen^1,3^, Ann Charis Tan^2^, Chia-Hao Chan^2^, Shuo-Ming Ou^1,2^, Fan-Yu Chen^1,2^, Ko-Wen Yu^1^, Yung-Tai Chen^1,4*^, Chih-Ching Lin^1,2*^

**Institutions:**

^1^School of Medicine, National Yang-Ming University, Taipei, Taiwan

^2^Division of Nephrology, Department of Medicine, Taipei Veterans General Hospital, Taipei, Taiwan

^3^Division of Nephrology, Department of Internal Medicine, National Yang-Ming University Hospital, Yilan, Taiwan

^4^Division of Nephrology, Department of Internal Medicine, Taipei City Hospital, Heping Fuyou Branch, Taipei, Taiwan

***Correspondence to:** Chih-Ching Lin, MD, PhD,

School of Medicine, National Yang-Ming University; Division of Nephrology, Department of Medicine, Taipei Veterans General Hospital,

No. 201, Section 2, Shih-Pai Road, Taipei 11217, Taiwan

**E-mail:** [lincc2@vghtpe.gov.tw](mailto:lincc2@vghtpe.gov.tw)

*Chih-Ching Lin and Yung-Tai Chen contributed equally to this work as corresponding authors.

| **Supplementary Table S1. Propensity Score Model Results of the Probability of ADPKD Diagnosis** | | | | | |
| --- | --- | --- | --- | --- | --- |
| **Parameter** | **Estimate** | **Odds Ratios** | **95% CI** | | |
|  |  |  | **Lower** | **Upper** | **P** |
| Age, per year | -0.0254 | 0.975 | 0.969 | 0.981 | <.0001 |
| Year of index date |  |  |  |  |  |
| 2000 |  | 1 |  |  |  |
| 2001 | 0.3507 | 1.42 | 0.855 | 2.358 | 0.1752 |
| 2002 | 0.1527 | 1.165 | 0.673 | 2.016 | 0.5852 |
| 2003 | 0.7717 | 2.163 | 1.336 | 3.502 | 0.0017 |
| 2004 | 0.5805 | 1.787 | 1.08 | 2.956 | 0.0238 |
| 2005 | 1.1257 | 3.082 | 1.936 | 4.908 | <0.0001 |
| 2006 | 1.1702 | 3.223 | 2.006 | 5.179 | <0.0001 |
| 2007 | 0.8627 | 2.37 | 1.429 | 3.928 | 0.0008 |
| 2008 | 1.1175 | 3.057 | 1.885 | 4.958 | <0.0001 |
| 2009 | 1.3891 | 4.011 | 2.505 | 6.423 | <0.0001 |
| 2010 | 1.2677 | 3.553 | 2.195 | 5.75 | <0.0001 |
| 2011 | 1.3017 | 3.675 | 2.239 | 6.033 | <0.0001 |
| Month of index date |  |  |  |  |  |
| January |  | 1 |  |  |  |
| February | 0.0113 | 1.011 | 0.681 | 1.502 | 0.9555 |
| March | -0.1091 | 0.897 | 0.603 | 1.334 | 0.5904 |
| April | 0.0273 | 1.028 | 0.691 | 1.529 | 0.893 |
| May | 0.2798 | 1.323 | 0.906 | 1.932 | 0.1477 |
| June | -0.0869 | 0.917 | 0.603 | 1.393 | 0.6842 |
| July | 0.1228 | 1.131 | 0.753 | 1.698 | 0.5539 |
| August | -0.1695 | 0.844 | 0.543 | 1.313 | 0.4519 |
| September | 0.2319 | 1.261 | 0.846 | 1.881 | 0.2553 |
| October | 0.058 | 1.06 | 0.703 | 1.598 | 0.7821 |
| November | -0.1404 | 0.869 | 0.563 | 1.341 | 0.5261 |
| December | -0.0567 | 0.945 | 0.625 | 1.429 | 0.788 |
| Male | -0.1374 | 0.872 | 0.731 | 1.039 | 0.1251 |
| Monthly income |  |  |  |  |  |
| Dependent |  | 1 |  |  |  |
| 0–19,100 NT$ | -0.0714 | 0.931 | 0.715 | 1.213 | 0.5973 |
| 19,100–42,000 NT$ | 0.1821 | 1.2 | 0.972 | 1.48 | 0.0894 |
| >NT 42,000 NT$ | 0.8667 | 2.379 | 1.7 | 3.329 | <0.0001 |
| Urbanization level^a^ |  |  |  |  |  |
| 1 |  | 1 |  |  |  |
| 2 | -0.0586 | 0.943 | 0.789 | 1.127 | 0.52 |
| 3 | -0.326 | 0.722 | 0.458 | 1.137 | 0.16 |
| 4 | -0.0462 | 0.955 | 0.389 | 2.345 | 0.9198 |
| CCI score | -0.2189 | 0.803 | 0.741 | 0.871 | <0.0001 |
| AVF | 0.1698 | 1.185 | 0.877 | 1.601 | 0.2681 |
| Concomitant medications |  |  |  |  |  |
| Antiplatelet agents^‡^ | -0.3674 | 0.693 | 0.567 | 0.846 | 0.0003 |
| ACE inhibitor or ARB | 0.2599 | 1.297 | 1.085 | 1.55 | 0.0043 |
| Beta blocker | 0.3988 | 1.49 | 1.24 | 1.79 | <0.0001 |
| Calcium channel blocker | 0.2758 | 1.318 | 1.074 | 1.617 | 0.0083 |
| Statin | -0.4042 | 0.668 | 0.474 | 0.941 | 0.0209 |
| Comorbidities |  |  |  |  |  |
| Diabetes mellitus | -0.267 | 0.766 | 0.59 | 0.993 | 0.0443 |
| Hypertension | -0.267 | 0.766 | 0.59 | 0.993 | 0.0443 |
| Myocardial infarction | 1.0155 | 2.761 | 1.817 | 4.195 | <0.0001 |
| Heart failure | 0.2584 | 1.295 | 0.832 | 2.014 | 0.2517 |
| Peripheral vascular disease | -0.3985 | 0.671 | 0.525 | 0.859 | 0.0016 |
| Dementia | 0.0782 | 1.081 | 0.732 | 1.597 | 0.6942 |
| Chronic pulmonary disease | 0.2383 | 1.269 | 0.74 | 2.176 | 0.3865 |
| Dyslipidemia | 0.3246 | 1.384 | 1.127 | 1.698 | 0.0019 |
| Cerebrovascular disease | -0.175 | 0.839 | 0.693 | 1.017 | 0.0741 |
| Valvular heart disease | 0.6067 | 1.834 | 1.454 | 2.314 | <0.0001 |
| Cancer | -0.1627 | 0.85 | 0.634 | 1.139 | 0.2764 |
| ^a^Urbanization levels in Taiwan are divided into four strata according to the Taiwan National Health Research Institute publications. Level 1 designates the most urbanized areas, and level 4 designates the least urbanized areas.  ^b^Charlson Comorbidity Index (CCI) score is used to determine overall systemic health. With each increased level of CCI score, there are stepwise increases in the cumulative mortality.[_._](file:///E:\Data%20D%20VGH\Scientific%20Reports\REVISION%232%20YTC\FINAL\Prognosis%20of%20vascular%20access%20in%20HD%20patients%20with%20ADPKD.docx#_ENREF_9)  ^‡^Including aspirin, clopidogrel, ticlopidine, and cilostazol  Abbreviations: ADPKD, autosomal dominant polycystic kidney disease; NT$, New Taiwan dollars; CCI, Charlson Comorbidity Index; AVF, arteriovenous fistula; ACE, angiotensin-converting enzyme; ARB, angiotensin II receptor blocker | | | | | |
